# Out of Plumb: When Water Treatment Causes Lead Contamination

**DOI:** 10.1289/ehp.117-a542

**Published:** 2009-12

**Authors:** Rebecca Renner

**Affiliations:** **Rebecca Renner**, PhD, of Williamsport, Pennsylvania, is a long-time contributor to *EHP* and *Environmental Science & Technology*. Her work has also appeared in *Scientific American*, *Science*, and Salon.com

In September 2009, parents, school staff, and politicians were unsettled to learn that children in many U.S. schools are quaffing not just water but lead and other contaminants when they quench their thirst at the school drinking fountain. But the distressing picture painted by the Associated Press analysis of data from the U.S. Environmental Protection Agency (EPA) may be just a small part of a more troubling whole, because the problem of lead in drinking water affects not only schools but homes. In recent years contamination with lead has emerged as an unintended consequence of water treatment changes aimed at improving water quality.

Because lead typically gets into drinking water only after it leaves the water treatment plant, it is difficult to monitor. “It is impossible to say how common or significant such exposures to lead and other metals are because contamination that occurs within the distribution system isn’t monitored,” says Rich Valentine, a professor of engineering at the University of Iowa.

According to the EPA, exposure to lead in drinking water can result in delayed physical or neurologic development in infants and children, and can cause high blood pressure, kidney problems, and cancer in adults. Moreover, a growing body of evidence suggests adverse health effects result even at blood levels below the 10 μg/dL “level of concern” at which the Centers for Disease Control and Prevention (CDC) recommends intervention.

“Lead in water is an underappreciated source of lead intake,” says Bruce Lanphear, a pediatric epidemiologist at Simon Fraser University in Vancouver. According to estimates from the CDC, on average drinking water makes up about 10–20% of children’s exposure to lead. Although paint and dust are the most common causes of elevated blood lead in children, in some cases tap water can be a primary source of exposure.

## Tap Water Linked to Excessive Lead

Drinking water typically starts out virtually free of lead because most source waters naturally have very low levels. The metal is introduced into tap water as it passes through lead service lines and across lead-soldered joints or as it sits next to brass and bronze fixtures that contain lead. In recent years lead contamination in tap water has been triggered by treatment changes that alter the water chemistry, destabilizing lead-bearing mineral scales that coated lead service lines and corroding lead-bearing solder, pipes, faucets, and fixtures.

Residents of Washington, DC, unwittingly drank water contaminated with lead from 2001 to 2004 when a switch in water disinfectant from chlorine to chloramine caused the release of lead. Water company monitoring records cited by the 31 January 2004 *Washington Post* showed that more than 4,000 homes tested had tap water lead levels above 15 ppb—the EPA action level at which utilities must take steps to remedy the problem. Hundreds had lead levels above 300 ppb; in a few homes and 1 school, the water from the tap contained more than 5,000 ppb lead.

In a study published in the 1 March 2009 issue of *Environmental Science & Technology*, Marc Edwards, an environmental engineer at Virginia Polytechnic Institute and State University, and Dana Best, a pediatrician at Children’s National Medical Center in Washington, DC, compared the number of DC infants and toddlers with blood lead levels greater than 10 μg/dL before and after the change in water treatment method. “At an absolute minimum this massive contamination affected many hundreds of kids for three years,” Edwards says. In addition, the 40,000 DC children who were either in the womb or using formula during the 3-year period may also have been affected by exposure to lead in tap water. These children, now 4–9 years old, may be at increased risk for future health and behavioral problems associated with lead exposure, according to Edwards. These findings contradict earlier studies that failed to find a link between DC tap water and children’s blood lead levels greater than 10 μg/dL.

Best says the findings changed her view of lead in water. “I was very surprised to see our results and find that lead-contaminated water can cause lead poisoning in kids,” she says. “I thought that lead in water was a problem of the past.”

But there is some evidence that it is not just a problem of the past and that DC is not unique, says Edwards. In Greenville, North Carolina, public health workers traced a 1-year-old boy’s blood lead levels of more than 20 μg/dL to tap water that had corroded lead solder in the home plumbing. In some cases, food such as pasta cooked in the contaminated water had become laced with minute particles of lead. Tests conducted on this pasta revealed that a single serving contained more lead than a dime-size lead paint chip. When the family stopped using contaminated tap water for cooking, the child’s blood lead level decreased dramatically.

Pitt County public health director John Morrow says, “I would like to know how common it is for lead in drinking water to elevate blood lead levels. We’ve tried to get parents to bring in their kids. We’ve tried to get doctors to test all 1- and 2-year-olds. But we’ve only tested about 45% of the kids [in the county], so we just don’t know.”

In 2006, tap water in Durham, North Carolina, was the source of elevated blood lead in another child. Public health officials linked the child’s poisoning to drinking water after they found more than 800 ppb lead in his tap water as a result of corroded solder. No other sources of lead were found in the child’s home.

Similarly, according to Greenville Utilities Commission plants manager Barrett Lassiter, there are no lead pipes in that city. Yet both the Durham and Greenville cases were ultimately attributed to a change in the coagulant chemical used by the cities’ utilities to clear the water of its natural turbidity. The change from alum to ferric chloride altered the chloride:sulfate ratio of the drinking water and also caused corrosion, Edwards says.

In Lakehurst Acres, a public housing development in Maine, a new anion exchange water treatment system that removes arsenic caused lead levels at the tap to exceed 1,000 ppb and resulted in elevated blood lead levels in several children and adults. Of 36 adults and children tested, 6 had blood lead levels equal to or greater than 10 μg/dL and 9 had levels of 5–9 μg/dL, according to Maine state toxicologist Andrew Smith.

Smith’s department traced a similar problem to at least 2 state schools that get their water from their own wells. Although well water typically is low in lead, it is often contaminated with naturally occurring arsenic. “[Anion exchange] is a popular way to remove arsenic,” says Smith. “I wonder how many others know that these arsenic removal systems can have unintended consequences on water chemistry that in turn can potentially release substantial amounts of lead long ago made relatively immobile.”

Environmental scientist Marie Lynn Miranda, director of the Children’s Environmental Health Initiative at Duke University, and colleagues also found an association between a change in water treatment and an increase in children’s blood lead levels in Wayne County, a third North Carolina locale. Their study, published in the February 2007 issue of *EHP*, looked at the effect of chloramine use. When the authors compared data on blood lead screening, housing age, and drinking water source for several thousand children, they found that the switch to chloramine coincided with an increase in elevated blood lead levels. The effect was more notable in houses built before 1950, which the authors say are more likely to have lead pipes or lead solder.

“Our work and that of teams like Edwards’ could change the way the public health community sees risks from water-borne lead and should focus attention on federal water regulations for lead, which are in sore need of revision,” says Miranda.

## LCR Loopholes

The EPA law that regulates lead in drinking water—the 1991 Lead and Copper Rule (LCR)—requires water companies to sample lead levels in home tap water. Private wells that serve day care centers, schools, or commercial enterprises also are covered under the rule. Water utilities must conduct sampling in a relatively small number of homes at high risk for elevated lead levels—for example, homes known to have lead service lines or lead solder. The size of the water system determines how many samples must be collected in each sampling period. For a major metropolis this could be 100 homes. For a system serving 10,000 homes or less, 40 samples must be collected. The sampling interval can vary from 6 months to 3 years; systems with good compliance sample less often.

The EPA requires water utilities to test the first flush (or first draw)—water that has stood in pipes for a minimum of 6 hours. Ideally there will be no lead in any sample, but under the LCR up to 10% of the high-risk households sampled may have lead levels that exceed 15 ppb. If more than 10% of this sample pool has tap water with lead levels exceeding 15 ppb, then utilities are required to notify customers and sometimes take remedial action, which can include replacing lead pipes that occur beneath publicly owned spaces such as streets and sidewalks.

“Most people think the current EPA standards for lead in drinking water are set to protect public health, says Yanna Lambrinidou, president of Parents for Nontoxic Alternatives, an children’s health advocacy group in Washington, DC. “So if a water utility says its water meets the lead standard, then people accept this and don’t worry about the water.” But essentially, a water company could meet all the EPA’s requirements and still have 9% of the homes sampled with hazardous levels of lead in their water.

Furthermore, there are myriad ways to miss high lead levels either accidentally or intentionally, says Edwards. These include failure to pick the worst-case houses, not allowing water to stand long enough before sampling, removing the aerator (a screen added to the faucet to reduce spray and/or conserve water) before sampling, and sampling in cooler months (when lead concentrations in water are lower because lead dissolves less readily in chilled water).

*Summary of Investigation Reported to the Board of Directors of the District of Columbia Water and Sewer Authority* [WASA], an independent 2004 report commissioned by WASA to investigate the causes of the DC lead contamination, lists numerous points where failure by the utility to follow best practices between 2001 and 2003 masked the scale of the problem. Five water samples with high lead levels were excluded, keeping WASA from exceeding the LCR limits. In some cases taps were flushed before sampling the water. And once the problem was recognized, the notice required by law to inform the public that excessive lead had been detected in tap water was printed as a small part of a glossy brochure about all sources of lead. According to the report, the brochure “did not clearly alert consumers that the recent spike in lead levels was a new reason for consumers to seriously consider the brochure’s educational content, nor did it convey that approximately half of the homes tested in the monitoring period had lead levels above 15 ppb.”

The report authors wrote that “WASA’s management made decisions to downplay some lead monitoring–related issues in its public communications.” Moreover, a “muted” response from the EPA and from other public agencies involved in water quality issues led to “missed opportunities to confront [lead exceedances] earlier.”

In response to the report, Glenn S. Gerstell, then chair of the WASA board of directors, released a statement acknowledging mistakes by the company but also criticizing the LCR. “It’s also obvious that the Lead and Copper Rule is ill-designed. The rule, and EPA’s enforcement of it, and consequently, WASA’s effort to comply with it, were focused on how to achieve a passing score, not how to inform the public and to truly and effectively address the underlying problem of lead levels in drinking water,” Gerstell wrote in the statement.

A difference in sampling procedures employed by the health department and the water company in Durham may partly explain how that city’s water passed EPA compliance monitoring and yet was responsible for a child’s lead poisoning. The tap with high lead values had an aerator that had collected lead solder particles. The flow of the water pushed the particles against the screen and shredded off tiny bits of lead much the way a grater shreds cheese, says Edwards. The health officials who identified the lead contamination sampled the tap water with the aerator on, just like people do when they dispense water into a cup or a pot. But the water company removed the aerator—and its load of lead—before collecting its sample for EPA compliance testing and so may have missed the high lead levels.

In October 2006 Stephen Heare, director of the EPA Drinking Water Protection Division, issued a memo to EPA Drinking Water Branch chiefs in Regions I through X directing that water companies “should not recommend that customers remove or clean aerators prior to or during the collection of tap samples for lead.” Prior to the 2006 memo, Heare acknowledged, the EPA had offered inconsistent advice about whether to remove the aerator.

## Working Together to Reveal the Extent of the Problem

It is not possible to say how many Americans may be drinking water contaminated with high levels of lead. However, according to a *Washington Post* investigation described in the 5 October 2004 issue of that paper, 274 U.S. utilities serving 11.5 million people reported high lead levels in drinking water in 2000–2004.

Like the *Post* investigation, outside studies consistently point to a widespread problem. The February 1993 issue of *Consumer Reports* reported that samples collected by several thousand readers revealed widespread lead contamination. In Chicago, where the building code required the use of lead water lines until 1986, *Consumer Reports* found 17% of first flush samples exceeded the LCR limit. The results contrasted with LCR compliance testing a year before that found just 3% over the limit. *Good Housekeeping* used a home inspection company to test drinking water in 8 metropolitan areas and reported in its 1 February 2005 issue that about 12% of the homes sampled overall had lead levels that exceeded government standards even though all of the cities were in compliance with the LCR.

These reports in popular publications make up the bulk of the literature on the state of affairs of lead in U.S. tap water. A 2006 Government Accountability Office report, *Drinking Water: EPA Should Strengthen Ongoing Efforts to Ensure that Consumers Are Protected from Lead Contamination*, concluded the EPA did not know the extent of lead contamination in drinking water supplies and needed to do more to ensure public protection. According to the report, EPA, state, and water system officials themselves identified 6 aspects of the LCR that would benefit from improved oversight: 1) ensuring that sampling sites reflect areas currently at highest risk, 2) deciding which water systems are eligible for less frequent monitoring, 3) informing homeowners who participate in monitoring of test results, 4) controlling when and how water treatment changes are implemented, 5) gathering data on the effectiveness of lead service line replacement programs, and 6) applying the LCR to water systems that sell drinking water to other systems.

Toward the end of 2007 the EPA strengthened the LCR in response to flaws exposed during the Washington, DC, crisis. For example, water companies are now required to seek approval from their oversight agency, usually the state, for significant water treatment changes.

Despite this action, lapses in compliance with the rule that were exposed in other cities have not been subject to enforcement actions, according to EPA insiders speaking on background only. The EPA Office of Water is exploring ways to further modify the LCR, such as prohibiting flushing the night before testing. A series of discussion white papers released by the agency last year indicates that some of the issues being considered include whether to add new water chemistry indicators to monitoring requirements and modifying advice on how water companies should select homes to monitor.

In response to the identification of water as the source of lead poisoning to children in the state, North Carolina has successfully brought together water companies, public health departments, and state regulators to address the issue of lead in drinking water. When water company sampling in compliance with the LCR yields a result that exceeds the EPA threshold, a copy of that result goes to the state health department, according to Ed Norman, an epidemiologist with the North Carolina Department of Environment & Natural Resources. Consequently, the state receives copies of several hundred water tests a year—these include day care centers, hotels, and restaurants if those premises happen to have been selected for LCR compliance monitoring. The state follows up with further sampling to establish the extent of the problem, the source of the lead, and how to fix it.

“The city water may be in compliance, and this may be one odd sample,” Norman says. “But it’s important to the individual property owner, and it’s important to the community when it’s a building that serves the public. We have state regulations covering food service and child care. We do what we can to fix the problem,” he says.

The recurrence of lead contamination of tap water indicates that more states need to implement such measures and that public health workers need to pay more attention to water as a source. “There is strong evidence that the problem of lead in drinking water is much bigger than realized,” says Edwards. “The preliminary information from schools, the emerging picture from Washington, DC, where hundreds of kids were lead poisoned, and a few cases from Maine and North Carolina where public health workers have been diligent enough to pursue the link between drinking water and lead poisoning to kids indicate that this is just the top of an iceberg.”

## Tips for Lead-Free Tap Water

Lead exposure is a serious concern for children’s health. Lead impairs children’s brain development, and many scientists believe no dose is safe. Because the law is not designed to monitor tap water lead levels in every individual home, people are ultimately on their own to ensure the safety of their drinking water.

Residents can have their tap water tested by their local health department. The EPA also provides links to state listings of certified water testing laboratories at http://www.epa.gov/safewater/labs/index.html. Parents can find out if their children’s school or day care center has tested each faucet for lead in the last few years and push schools to have them tested, especially if the locality’s water treatment process has changed significantly.

The EPA recommends cleaning home faucet aerators about once every 2 weeks and letting tap water run until it “becomes as cold as it will get” before drawing water for use, which can take 2 minutes or longer. The agency also recommends using only cold tap water for cooking, drinking, and preparing baby formula.

Pur^™^, Brita^™^, or ZeroWater^®^ pitcher filters can reduce dissolved lead and other metals. These products use a cation/anion exchange process. Brita and Pur faucet attachments have screens that can trap sediments and a compressed block of carbon and zeolite that captures contaminants as water flows through. The standard models of these products retail for less than $50 but require filter replacements.

Other filtration systems, which can be installed at the sink, use reverse osmosis to remove lead and other contaminants from tap water. These systems typically cost in the hundreds of dollars and operate by passing water through a semipermeable membrane that traps contaminants.

Consumers should make sure the filter they select is certified to comply with the National Sanitation Foundation/American National Standards Institute (NSF/ANSI) Standard 53 for drinking water treatment units; for reverse osmosis systems, NSF/ANSI 58 is the applicable standard. Certification verifies that a water sample was independently tested to verify the treatment system could reduce lead to 0.010 mg/L or less.

Water distilling systems also remove lead and other contaminants from water. These come in portable and countertop models and also run into the hundreds of dollars in cost. Water distillers separate water from contaminants using evaporation and condensation.

## Figures and Tables

**Figure:**
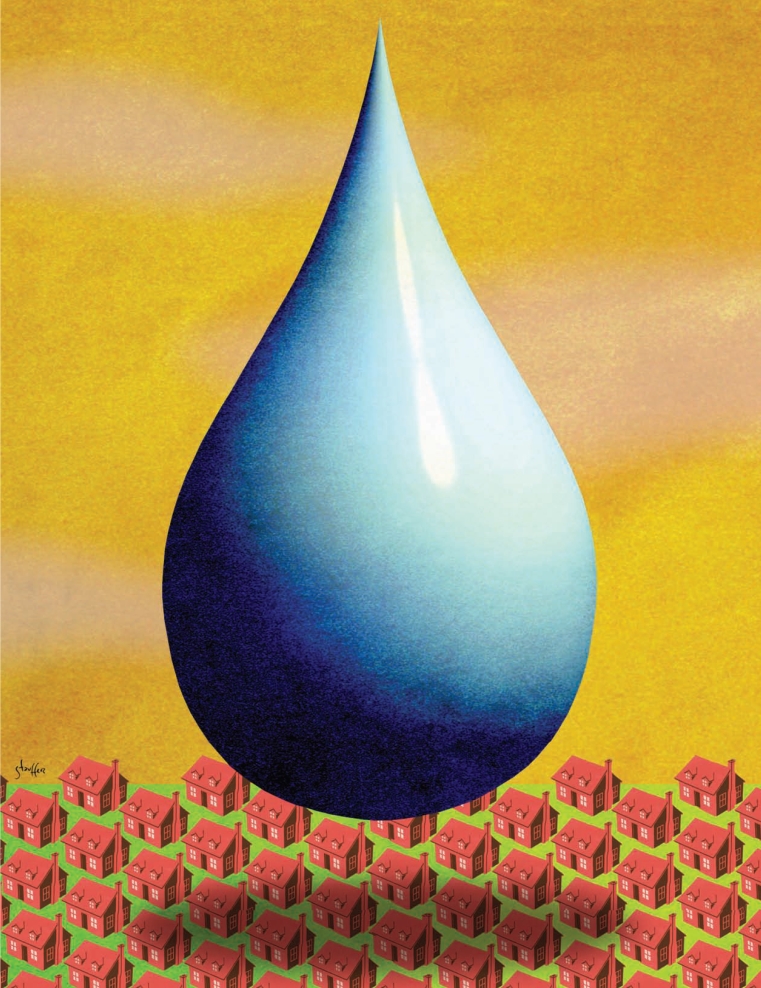


**Figure:**
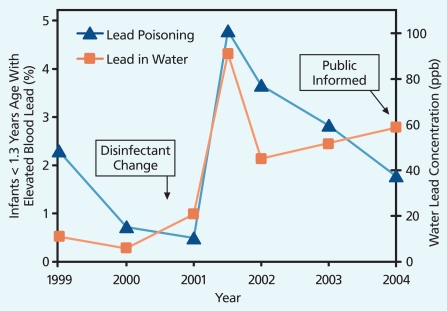
**A retrospective analysis of water utility data on lead in drinking water and data on child blood levels in Washington, DC, revealed that a spike in the number of infants with blood lead levels of 10 μg/dL or higher correlated strongly with a change in the city’s water treatment.** Source: Marc Edwards. Adapted from Edwards M et al. 2009. Elevated blood lead in young children due to lead-contaminated drinking water: Washington, DC, 2001–2004. Environ Sci Technol 43(5):1618–1623.

**Figure:**
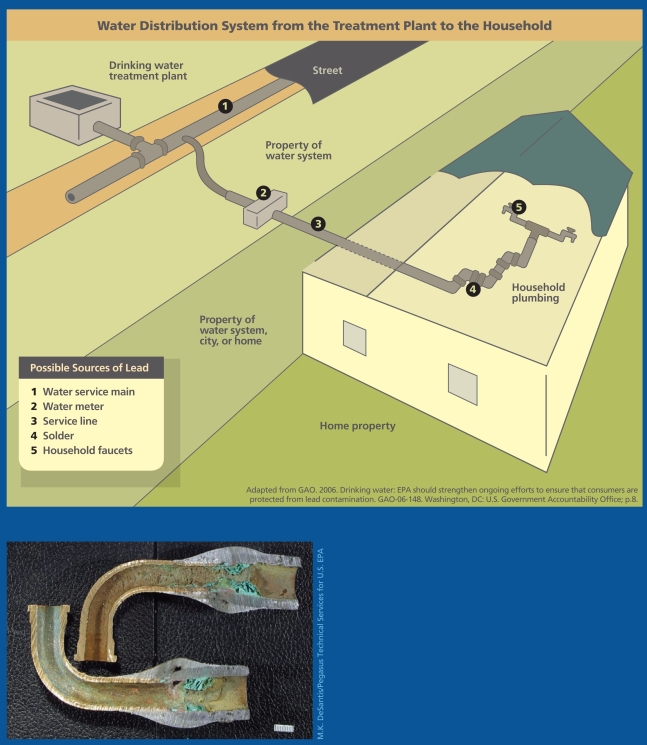
Deposits of lead, copper, and other minerals can form inside plumbing pipes when the pipe walls corrode through oxidation or other chemical action. Corrosion deposits and mineral scale can serve as reservoirs for the accumulation of contaminants in water, which can become destabilized with subsequent changes in water chemistry. Short sections of pipe known as “goosenecks” or “pigtails” (left) connect the water main to individuals’ service lines. Although the service lines themselves may be lead-free, in many cities these goosenecks are sources of pure lead. Moreover, the point where two different metals meet is often the site of galvanic corrosion.

